# Supplementary material for: Minimum recommended micronutrient intake status and associated factors among pastoralist children aged 6–23 months in Aysaita district, Afar Region, Ethiopia, 2024: A community based cross-sectional study
Source: PLoS One. 2025 Oct 28;20(10):e0334736. doi: 10.1371/journal.pone.0334736 (PMC12561942; doi:10.1371/journal.pone.0334736)
Supplement: S1 Questionaries — The questionnaire contains sections on socio-demographic and economic characteristics, obstetric characteristics of the mothers, child characteristics and common childhood illness and minimum recommended micronutrient intake which holds 11 questions. (PDF) [file pone.0334736.s001.pdf]

Appended I: English version questionnaires

Title: To assess minimum micronutrient intake status and associated factors among children aged 6–23 months in Aysaita district, Afar regional state, Ethiopia, 2024. ***Community based cross-sectional study.***

**Part-1. Socio demographic characteristics of the respondent**				
S. N	***Questions***	***Alternative responses***	***Skip***	***Code***
101	Age of mothers/caregivers in years	______		
102	Sex of a child	1. Male<br>2. Female		
103	Place of residence?	1. Rural<br>2. Urban		
104	Religion of mothers/caregivers in years	1. Orthodox<br>2. Protestant<br>3. Catholic<br>4. Muslim<br>5. Others____		
105	Ethnicity	1. Afar<br>2. Ahmara<br>3. Tigre<br>4. Oromo<br>5. Others		
106	Current marital status	1. Single<br>2. Married<br>3. Widowed<br>4. Divorced		
107	Educational status of mothers/caregiver's	1. Illiterate<br>2. Primary education<br>3. Secondary education		

		4. More than secondary		
108	Educational status of husband's/partner's	1. Illiterate<br>2. Primary education<br>3. Secondary education<br>4. More than secondary		
109	Occupation of the mothers/caregiver's	1. Employee<br>2. Housewife<br>3. Merchant<br>4. Farmer<br>5. Daily work<br>6. Others		
110	Occupation status of husband's/partner's	1. Employee<br>2. Merchant<br>3. Farmer<br>4. Daily work<br>5. Others		
111	Family size	1. ≤ 4<br>2. >4		
112	Monthly family income (in Ethiopian Birr)?	________________		
**Part-2: Obstetric characteristics of the mothers/care givers.**				
201	Age at 1st pregnancy	_______		
202	Antenatal care follow-up status	0. No antenatal care follow up<br>1. Have antenatal care follow up<br>2. Don't know		
203	Desire for more children	1. Wants<br>2. Undecided		

		3. Wants no more		
204	Place of delivery	1. Health facility<br>0. Home		
205	Mode of delivery	1. Spontaneous vaginal delivery<br>0. Cesarean section		
206	PNC check up	1. Yes<br>0. No		
207	Exclusive breastfeeding status	1. Yes<br>0. No		
208	Current pregnancy status	1. Pregnant<br>0. Non-pregnant or unsure		
209	Media exposure	1. Yes<br>0. No		
**Part-3: Child characteristics and common childhood illness**				
301	Current age of the child (months)	________		
302	Had diarrhea in the past two weeks	1. Yes<br>0. No		
303	Had cough in the past two weeks	1. Yes<br>0. No		
304	Had fever in the past two weeks	1. Yes<br>0. No		
**Part-4: Micronutrient intake status among children aged 6–23 months**				
	Food groups and supplementations	***Contains/measurements***	***Response***	
401		Eggs	0. No<br>1. Yes	
402		Meat (beef, lamb, chicken, etc)	0. No<br>1. Yes	
403		Pumpkin, carrots, and squash	0. No<br>1. Yes	

404	***Consumed foods rich in vitamin A within 24 hours***	Any dark green leafy vegetables	0. No<br>1. Yes	
405		Mangoes, papayas, and others with VA fruits	0. No<br>1. Yes	
406		Liver, heart, and other organs	0. No<br>1. Yes	
407		Fish or shellfish.	0. No<br>1. Yes	
408	***Consumed foods rich in iron at any time in 24 hours***	Eggs	0. No<br>1. Yes	
409		Meat (beef, lamb, chicken)	0. No<br>1. Yes	
410		Liver, heart, and other organs	0. No<br>1. Yes	
411		Fish or shellfish	0. No<br>1. Yes	
412	Multiple micronutrient powder in the past seven days		0. No<br>1. Yes	
413	Iron supplements within seven days (iron pills, sprinkles with iron, or iron syrup in the previous seven days)		0. No<br>1. Yes	
414	Vitamin A supplementation within six months (by reviewing the integrated child health card or immunization card or growth monitoring history) otherwise from the mother’s verbal response.		0. No<br>1. Yes	
415	Deworming medication in the six months (by reviewing the integrated child health card or immunization card or growth monitoring history) otherwise from the mother’s verbal response.		0. No<br>1. Yes	
