# Supplementary material for: Minimum recommended micronutrient intake status and associated factors among pastoralist children aged 6–23 months in Aysaita district, Afar Region, Ethiopia, 2024: A community based cross-sectional study
Source: PLoS One. 2025 Oct 28;20(10):e0334736. doi: 10.1371/journal.pone.0334736 (PMC12561942; doi:10.1371/journal.pone.0334736)
Supplement: Attachment — Submitted filename: PONE-D-24-55242_reviewed document.pdf [file pone.0334736.s003.pdf]

PLOS ONE

Minimum Recommended Micronutrient Intake Status and Associated Factors among Children Aged 6-23 Months in Pastoralist Community of Afar Region, Ethiopia, 2024 : Community Based Cross-sectional Study

--Manuscript Draft--

**Manuscript Number:**	PONE-D-24-55242
**Article Type:**	Research Article
**Full Title:**	Minimum Recommended Micronutrient Intake Status and Associated Factors among Children Aged 6-23 Months in Pastoralist Community of Afar Region, Ethiopia, 2024 : Community Based Cross-sectional Study
**Short Title:**	Minimum Recommended Micronutrient Intake Status among children Aged 6-23 Months in Pastoralist Community.
**Corresponding Author:**	Abdulkerim Hassen Moloro, MSc<br>Samara University<br>Semera, Afar ETHIOPIA
**Keywords:**	Key words: Minimum, Micronutrient, Children aged 6-23 months, Pastoralist, Afar, Ethiopia
**Abstract:**	Abstract<br><br>Background: Micronutrient deficiencies are among the most prevalent public health problems in developing countries like Ethiopia. Despite government efforts to address these deficiencies, progress remains slow, leaving many children to endure serious health consequences. There is a scarcity of studies examining the minimum recommended micronutrient intake among children aged 6-23 months in this region.<br>Objective: This study aimed to assess minimum recommended micronutrient intake status and associated factors among children aged 6-23 months in pastoralist community of Afar Region, Ethiopia, 2024.<br>Methods: A community-based cross-sectional study design was conducted among 675 children aged 6-23 months with mothers from August 1-30/2024. Multi-stage sampling technique was used to select study participants. Data were collected by KoboToolbox using an interview-based structured questionnaire. The collected data were exported to Excel and then into STATA version 17 software packages. Descriptive statistics such as proportion, mean, median, cross-tabulation, and frequencies were calculated and presented in tables. Bivariable and multivariable logistic regression analyses at a 95% confidence interval (CI) and a P-value of less than 0.05 in the multivariable logistic regression were considered significant predictors.<br>Results: All study participants were included for data collection by giving a response rate of 100%. In this study, only 34.96% of the children aged 6-23 months had received at least one minimum recommended micronutrient intake status (34.96%; 95% CI: 31.45, 38.64%). Being a male child (AOR: 1.98, 95% CI: 1.41, 2.78), having media exposure (AOR = 2.09; 95% CI: 1.16, 3.75), having exclusive breast feeding (AOR: 4.53, 95% CI: 2.51, 8.19), being in the income category 500-5000 (AOR: 1.99, 95% CI: 1.17, 3.39) and spontaneous vaginal delivery (AOR: 2.42, 95% CI: 1.12, 5.25) were factors associated with the minimum recommended micronutrient intake status.<br>Conclusions and Recommendations: The study revealed over one-third of the children received the minimum recommended micronutrient intake. Factors such as being male, media exposure, exclusive breastfeeding, spontaneous vaginal delivery, and household income were significantly associated with achieving the minimum recommended micronutrient intake. This shortfall indicates that the targets (improving the nutritional status of women (15-49 years) and adolescent girls (10-19 years and enhancing the nutritional status of women from childbirth up to 10 years) set by the National Nutrition Program (NNP II) 2016-2020 have not been met. Immediate and targeted interventions like expanding access to micronutrient supplementation (such as vitamin A, iron, and folic acid) for children and pregnant women are essential to meet the National Nutrition Program (NNP II) 2016-2020 targets in the study area.
**Order of Authors:**	Ali Mohammed Ibrahim, MSc

	Abdulkerim Hassen Moloro, MSc
	1Dereje Desalegn Boshe
	Yeshimebet Ali Dawed
**Additional Information:**	
**Question**	**Response**
**Financial Disclosure**<br><br>Enter a financial disclosure statement that describes the sources of funding for the work included in this submission. Review the submission guidelines for detailed requirements. View published research articles from *PLOS ONE* for specific examples.<br><br>This statement is required for submission and **will appear in the published article** if the submission is accepted. Please make sure it is accurate.<br><br>**Funded studies**<br>Enter a statement with the following details:<br>• Initials of the authors who received each award<br>• Grant numbers awarded to each author<br>• The full name of each funder<br>• URL of each funder website<br>• Did the sponsors or funders play any role in the study design, data collection and analysis, decision to publish, or preparation of the manuscript?<br><br>Did you receive funding for this work?	The author(s) received no specific funding for this work.
**Competing Interests**<br><br>Use the instructions below to enter a competing interest statement for this submission. On behalf of all authors, disclose any competing interests that could be perceived to bias this work—acknowledging all financial support and any other relevant financial or non-financial competing interests.<br><br>This statement is required for submission	The authors have declared that no competing interests exist.

and **will appear in the published article** if the submission is accepted. Please make sure it is accurate and that any funding sources listed in your Funding Information later in the submission form are also declared in your Financial Disclosure statement.<br><br>View published research articles from *PLOS ONE* for specific examples.<br><br>**NO authors have competing interests**<br><br>Enter: *The authors have declared that no competing interests exist.*<br><br>**Authors with competing interests**<br><br>Enter competing interest details beginning with this statement:<br><br>*I have read the journal's policy and the authors of this manuscript have the following competing interests: [insert competing interests here]*<br><br>* typeset	
**Ethics Statement**<br><br>Enter an ethics statement for this submission. This statement is required if the study involved:<br><br>• Human participants<br>• Human specimens or tissue<br>• Vertebrate animals or cephalopods<br>• Vertebrate embryos or tissues<br>• Field research<br><br>Write "N/A" if the submission does not require an ethics statement.<br><br>General guidance is provided below. Consult the submission guidelines for detailed instructions. **Make sure that all information entered here is included in the Methods section of the manuscript.**	Ethical Statements<br>Ethical approval was obtained from the Institutional Ethical Review Committee (IERC) of Samara University, College of Medicine and Health Science, with reference number (Ref. No.: SU/IRB/814/2016). Following the approval by IERC, official letters of cooperation were written to the Aysaita district health administration office, and in turn, the woreda health administration office wrote letters to each selected kebeles and village's administration office in order to get permission and cooperation. The oral informed consent from the respondent was obtained after the purpose and objective of the study explained. To ensure confidentiality, the names of respondents were replaced by code numbers. Participants were given the chance to ask any doubt about the study and made free to refuse or stop the interview at any moment they wanted.

**Format for specific study types**<br><br>**Human Subject Research (involving human participants and/or tissue)**<br>• Give the name of the institutional review board or ethics committee that approved the study<br>• Include the approval number and/or a statement indicating approval of this research<br>• Indicate the form of consent obtained (written/oral) or the reason that consent was not obtained (e.g. the data were analyzed anonymously)<br><br>**Animal Research (involving vertebrate animals, embryos or tissues)**<br>• Provide the name of the Institutional Animal Care and Use Committee (IACUC) or other relevant ethics board that reviewed the study protocol, and indicate whether they approved this research or granted a formal waiver of ethical approval<br>• Include an approval number if one was obtained<br>• If the study involved *non-human primates*, add *additional details* about animal welfare and steps taken to ameliorate suffering<br>• If anesthesia, euthanasia, or any kind of animal sacrifice is part of the study, include briefly which substances and/or methods were applied<br><br>**Field Research**<br><br>Include the following details if this study involves the collection of plant, animal, or other materials from a natural setting:<br>• Field permit number<br>• Name of the institution or relevant body that granted permission	
**Data Availability**<br><br>Authors are required to make all data underlying the findings described fully available, without restriction, and from the time of publication. PLOS allows rare exceptions to address legal and ethical concerns. See the PLOS Data Policy and FAQ for detailed information.	Yes - all data are fully available without restriction

Powered by Editorial Manager® and ProduXion Manager® from Aries Systems Corporation

A Data Availability Statement describing where the data can be found is required at submission. Your answers to this question constitute the Data Availability Statement and **will be published in the article**, if accepted.<br><br>**Important:** Stating 'data available on request from the author' is not sufficient. If your data are only available upon request, select 'No' for the first question and explain your exceptional situation in the text box.<br><br>Do the authors confirm that all data underlying the findings described in their manuscript are fully available without restriction?	
**Describe where the data may be found in full sentences. If you are copying our sample text, replace any instances of XXX with the appropriate details.**<br><br>• If the data are **held or will be held in a public repository**, include URLs, accession numbers or DOIs. If this information will only be available after acceptance, indicate this by ticking the box below. For example: *All XXX files are available from the XXX database (accession number(s) XXX, XXX.*).<br>• If the data are all contained **within the manuscript and/or Supporting Information files**, enter the following: *All relevant data are within the manuscript and its Supporting Information files.*<br>• If neither of these applies but you are able to provide **details of access elsewhere**, with or without limitations, please do so. For example:<br><br>*Data cannot be shared publicly because of [XXX]. Data are available from the XXX Institutional Data Access / Ethics Committee (contact via XXX) for researchers who meet the criteria for access to confidential data.*<br><br>*The data underlying the results presented in the study are available from (include the name of the third party*	All data are available based on the reasonable request from principal investigator or corresponding author

and contact information or URL).<br>• This text is appropriate if the data are owned by a third party and authors do not have permission to share the data.<br><br>* typeset	
Additional data availability information:	

Powered by Editorial Manager® and ProduXion Manager® from Aries Systems Corporation

Revised Manuscript
Click here to access/download;Manuscript;Micronutrient intake manuscript revised 3.docx

Minimum Recommended Micronutrient Intake Status and Associated Factors among Childrens Aged 6-23 Months in Pastoralist Community of Afar Region, Ethiopia, 2024: Community Based Cross-sectional Study

Author's Name and Email

[1]Ali Mohammed Ibrahim, Email: Aliawu61@gmail.com

[2]Abdulkerim Hassen Moloro*, Email: habdulkerim4@gmail.com

[1]Dereje Desalegn Boshe, Email: djareka@gmail.com

[3]Yeshimebet Ali Dawed, Email: ymaali32@gmail.com

Authors Affiliation

[1]Department of Public Health, College of Medicine and Health Sciences, Samara University, Samara, Ethiopia

[2]Department of Nursing, College of Medicine and Health Sciences, Samara University, Samara, Ethiopia

[3]Department of Nutrition and Dietetics, School of Public Health, College of Medicine and Health Sciences, Wollo University, Dessie, Ethiopia

Corresponding Authors*

Abdulkerim Hassen Moloro*, BSc, MSc in Adult Health Nursing

Department of Nursing

College of Medicine and Health Science

Samara University

Samara,

Ethiopia

P.O. Box: 132

Phone: +251 935912095/+251952695335

Email: habdulkerim4@gmail.com

ORCID iD: https://orcid.org/0000-0002-6081-4108

Abstract

Background: Micronutrient deficiencies are among the most prevalent public health problems in developing countries like Ethiopia. Despite government efforts to address these deficiencies, progress remains slow, leaving many children to endure serious health consequences. There is a scarcity of studies examining the minimum recommended micronutrient intake among children aged 6-23 months in this region.

Objective: This study aimed to assess minimum recommended micronutrient intake status and associated factors among children aged 6-23 months in pastoralist community of Afar Region, Ethiopia, 2024.

Methods: A community-based cross-sectional study design was conducted among 675 children aged 6-23 months with mothers from August 1-30/2024. Multi-stage sampling technique was used to select study participants. Data were collected by KoboToolbox using an interview-based structured questionnaire. The collected data were exported to Excel and then into STATA version 17 software packages. Descriptive statistics such as proportion, mean, median, cross-tabulation, and frequencies were calculated and presented in tables. Bivariable and multivariable logistic regression analyses at a 95% confidence interval (CI) and a P-value of less than 0.05 in the multivariable logistic regression were considered significant predictors.

Results: All study participants were included for data collection by giving a response rate of 100%. In this study, only 34.96% of the children aged 6-23 months had received at least one minimum recommended micronutrient intake status (34.96%; 95% CI: 31.45, 38.64%). Being a male child (AOR: 1.98, 95% CI: 1.41, 2.78), having media exposure (AOR = 2.09; 95% CI: 1.16, 3.75), having exclusive breast feeding (AOR: 4.53, 95% CI: 2.51, 8.19), being in the income category 500-5000 (AOR: 1.99, 95% CI: 1.17, 3.39) and spontaneous vaginal delivery (AOR: 2.42, 95% CI: 1.12, 5.25) were factors associated with the minimum recommended micronutrient intake status.

Conclusions and Recommendations: The study revealed over one-third of the children received the minimum recommended micronutrient intake. Factors such as being male, media exposure, exclusive breastfeeding, spontaneous vaginal delivery, and household income were significantly associated with achieving the minimum recommended micronutrient intake. This shortfall indicates that the targets (improving the nutritional status of women (15-49 years) and adolescent girls (10-19 years and enhancing the nutritional status of women from childbirth up to 10 years) set by the National Nutrition Program (NNP II) 2016-2020 have not been met. Immediate and targeted interventions like expanding

access to micronutrient supplementation (such as vitamin A, iron, and folic acid) for children and pregnant women are essential to meet the National Nutrition Program (NNP II) 2016-2020 targets in the study area.

Key words: Minimum, Micronutrient, Children aged 6-23 months, Pastoralist, Afar, Ethiopia

1. Introduction

1.1 Background

The initial two years of life are a critical period for development, significantly influencing immune and physiological functions (1). However, during this time, children are highly susceptible to undernutrition due to factors like inadequate dietary intake, limited access to food, nutritional taboos, and infectious diseases (2). In Ethiopia, micronutrient deficiencies among children are a major public health issue(3). The most prevalent deficiencies include iron, iodine, vitamin A, and zinc (4). These deficiencies are responsible for over half of all deaths in children under five worldwide (5). Additionally, they contribute to weakened immunity, intellectual disabilities, reduced nutrient absorption, and delayed or impaired physical, mental, and psychomotor development (6-8).

Between 1990 and 2017, Ethiopia experienced a decrease in dietary deficiencies, with iron deficiency dropping by 20.1%, vitamin A deficiency by 16.7%, and iodine deficiency by 91.6% (9). The National Nutrition Program (NNP II) is dedicated to implementing nutrition interventions aimed at ending hunger by 2030, including efforts to address and prevent micronutrient deficiencies (10). Despite these governmental efforts, key indicators of micronutrient intake among young children remain low. For example, only 29% of children aged 6 to 23 months consumed vitamin A-rich foods, and 24% consumed iron-rich foods (11). Additionally, just 6% of children aged 6 to 59 months received iron supplementation, while 53% received vitamin A supplementation (11). Consequently, micronutrient deficiencies continue to be a significant issue in the country (9).

According to the 2016 Ethiopia Demographic and Health Survey (EDHS), only 14% of children aged 6 to 23 months met the Minimum Dietary Diversity (MDD) criteria (12). Additionally, the 2016 Ethiopian National Nutritional Supplementation Survey reported that vitamin A Supplementation (VAS) coverage among children was 63%, falling short of the national target of over 90% (13). The national prevalence of subclinical Vitamin A deficiency (serum retinol < 0.7 μmol/L) was alarmingly high at 37.7%(14, 15).

Micronutrient deficiencies are significant public health issues in Ethiopia, primarily due to diets lacking diversity, low bioavailability, frequent meal skipping, limited access to micronutrient-rich and fortified foods, and low intake of vegetables and fruits (16-18). To combat these deficiencies among children, the national nutritional supplementation program provides both food and supplements. For children older than six months, recommended micronutrients include foods rich in vitamin A and iron, multiple micronutrient powders, iron and vitamin A supplements (VAS), and deworming for those older than 12 months (19-21). VAS and deworming are administered semi-annually to children aged 6 to 59 months as part of the national nutrition program. For children aged 6 to 23 months, it is recommended that they consume four or more food groups rich in iron and vitamin A within the previous 24 hours, receive multiple micronutrient powders and iron supplementation within seven days, and have over 75% deworming coverage within the last six months. Interventions to improve maternal nutrition include multiple micronutrient supplements, food fortification, supplementary food, nutrition education, and counseling, primarily through community-based nutrition programs in Ethiopia (22).

Despite global efforts, progress towards the 2025 World Health Assembly's nutrition targets, particularly in reducing child wasting and stunting through adequate dietary intake, remains insufficient (23). Meeting the 2030 Sustainable Development Goals (SDGs), especially SDG 2, which aims to end hunger, achieve food security, and improve nutrition, also presents significant challenges(24). This lack of progress is most pronounced in low- and middle-income countries, including Ethiopia (25). The Ethiopian government has made notable progress in addressing various forms of malnutrition. To further this goal, it has developed a comprehensive Food and Nutrition Policy (FNP) (26). Additionally, the Seqota Declaration underscores the government's commitment to eradicating undernutrition by 2030 (27, 28). Although the Ethiopian government has implemented various measures, the fight against malnutrition is advancing slowly, leaving many children to suffer from its severe impacts (29-31).

While there is documented evidence of inadequate micronutrient intake among both agrarian communities and urban dwellers in Ethiopia (32, 33), there is limited information on the micronutrient intake of children aged 6–23 months in the Afar. These areas are predominantly inhabited by pastoralist communities with limited cultivation of fruits and vegetables (34). Furthermore, the Afar regions are recognized as hotspots for high food insecurity, elevated child malnutrition rates, poor infrastructure, inaccessible health and recurrent droughts (35-38). As far as literature searched, there is a scarcity of studies examining the individual and community-level minimum recommended micronutrient intake status and factors

influencing micronutrient intake among children aged 6-23 months in the study area. Therefore, this study aimed to evaluate the minimum recommended micronutrient intake status and associated factors among children aged 6–23 months in the pastoralist community of Afar region, Ethiopia.

2. Methods

2.1 Study area and period

Afar National Regional State is one of the eleven regional states of Ethiopia. Samara is the capital city of the Afar national regional state, which is 570 km from Addis Ababa and located in the north-eastern part of the country. The size of Afar National Regional State is 278,000 sq. km. Geographically, the region is located between 9°N and 12°N latitude and 40°E and 42°E longitude at the northern tip of the Great East African Rift Valley and is found at 432 m altitude. Afar regional state has six administrative zones, such as Awsi Rasu (zone one), Kilbat Rasu (zone two), Gabi Rasu (zone three), Fanti Rasu (zone four), Hari Rasu (zone five), and Yagudae Rasu (zone six).

Based on the 2007 Census conducted by the Central Statistical Agency of Ethiopia, the region has a population of 1,390,273, consisting of 775,117 men and 615,156 women, and a total population projection for 2017 was 1,812,002, consisting of 991,000 men and 821,002 women(39). The majority of the population has a pastoral lifestyle, but there are still agrarian and agro-pastoral societies in the region.

For this study, Assayta district was selected randomly, which was found in Awsi Rasu (Administrative Zone 1) within the Afar Region, Ethiopia. It is located 600 km northeast of Addis Ababa and 72 km away from Samara town. The district is administratively divided into 11 kebeles. According to the Aysaita District Administration, the total human population of the district is 61961. Among the total population of the district, 12392 households have children aged 6–23 months. The study was conducted from August 1-30, 2024.

2.2 Study design

A community-based cross-sectional study was conducted Study design was used

2.3 Population

2.3.1 Source population

All children aged 6-23 months and mothers/caregivers residing in pastoralist communities of the Afar region were source populations.

2.3.2 Study population

All children 6-23 months old and who are residents of the selected kebeles in the selected district.

2.4 Eligibility Criteria

2.4.1. Inclusion criteria

The study included all breastfed and non-breastfed children aged 6–23 months who had been living in the designated study area for at least 6 months before the survey and were residing with their biological mothers aged 18–49 years.

2.4.2. Exclusion criteria

Children living with others than their mothers or caregivers were excluded from the analysis. Children have severe acute malnutrition or chronic illnesses requiring specialized dietary management. Mothers who have a health problem that can affect the interview process.

2.5 Sample size determination and Sampling technique

2.5.1 Sample size determination

The sample size was determined by ***OpenEpi online software version 3.1*** using the single population proportion formula by considering the estimated proportion of micronutrient intake status, 59.1% in Ethiopia, from the study conducted in sub-Saharan Africa in 2023 (40), with a 95% confidence level and a 5% marginal error. As a result, **409** samples were obtained. Since it is a multistage sampling technique, using the design effect multiplied by 1.5 becomes 614, and by adding a 10% non-response rate, the final sample size becomes **675.**

2.5.2 Sampling procedure

A multi-stage sampling technique was employed to select the study participants. First, Zone One was randomly selected from the six zones in the Afar region. Next, Aysaita district was randomly chosen from Zone One. Then, five kebeles were randomly selected from the eleven kebeles in the district. The sample size allocated to each selected kebele was proportional to the number of children aged 6–23 months (Figure 1). The value of ‘K’ was calculated from N/n, where N represents the total number of children and n represents the sample size (K = N/n = 5803/675 = 8). Consequently, every eighth child from each kebele participated in the study. The expected total number of participants in the selected kebeles was n = 675. The study participants were selected using systematic sampling with a skip interval of 8 in each kebele. The sample frame of households (mothers who delivered in the last two years and have children

aged 6-23 months) was prepared in all selected kebeles from the mother's registration book, obtained from health extension workers. Additionally, a survey was conducted to identify a list of households (mothers who delivered in the last two years and have children aged 6–23 months) in case the household list was not registered in the kebeles before the actual data collection.

For mothers with twins, only one was selected by lottery methods. Subsequently, every 8th mother was included until the desired sample size was achieved. In households in which mothers or candidate respondents were not at home, but it was known that there were eligible households for the study, the interviewers revisited the household at three different time intervals, and no interviewers failed to meet the household, and the household was not replaced by the next household in a clockwise direction.

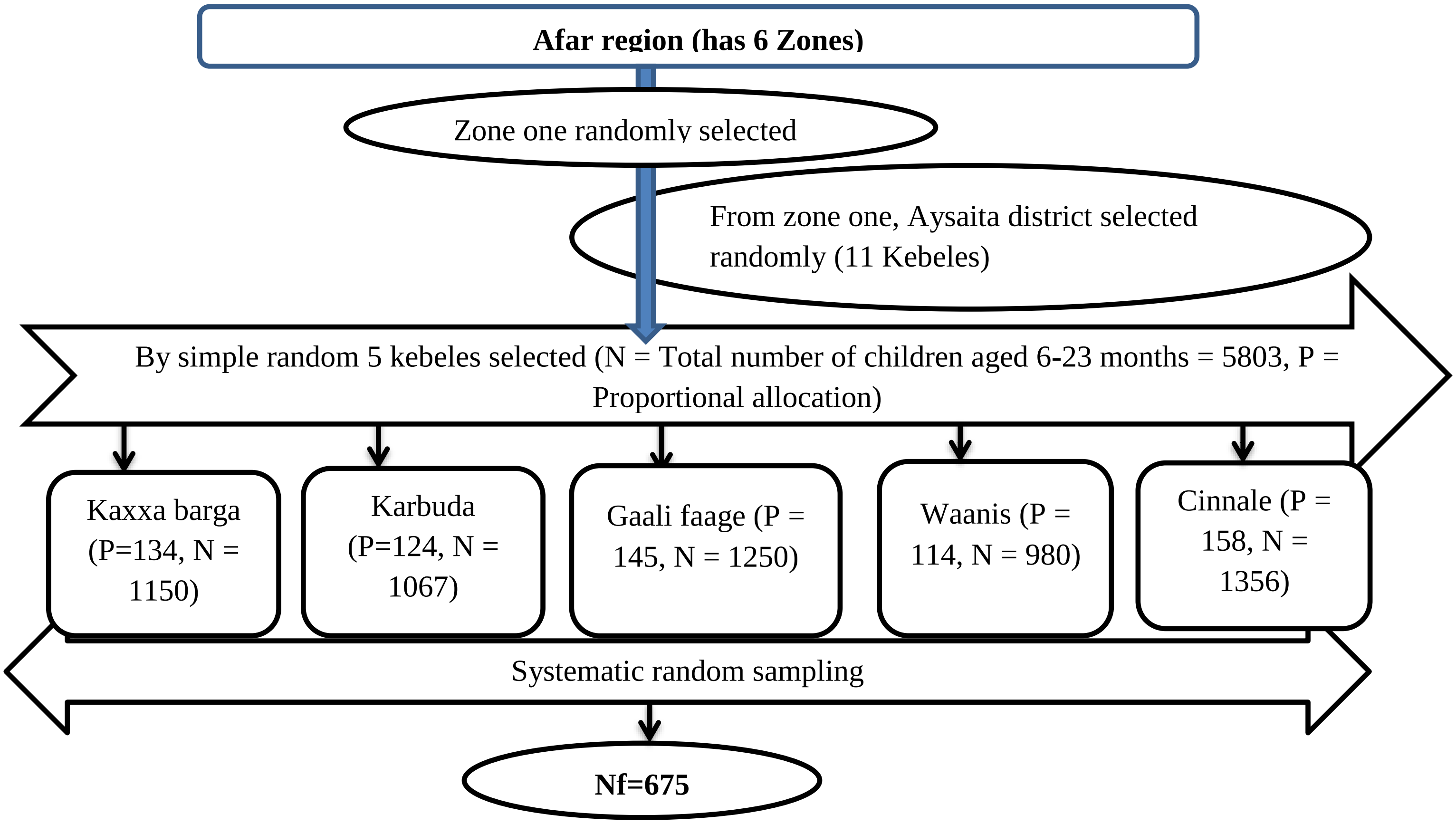

Figure 1: Schematic presentation of the sampling procedure of study participants in assessment of minimum recommended micronutrient intake status and associated factors among children aged 6-23 months in the pastoralist community, Afar region, Ethiopia, 2024.

4.6 Study Variable

4.6.1 Dependent variable

Minimum recommended micronutrient intake status

2.6.2 Independent variable

Socio-demographic and economic characteristics: Age of mothers, sex of the child, place of the residence, family size, ethnicity, religion, marital status, educational status, husband educational status, Occupational status, husband educational status, family monthly income (ETB), functional electricity, functional radio, functional television, functional refrigerator, and bank account.

Obstetric characteristics of the mothers: Age at first pregnancy, Antenatal care follow up, desire for more childrens, place of delivery, mode of delivery, postnatal check up, had exclusive breast feeding, current pregnancy status, and media exposure.

Child characteristics and common childhood illness: Child age in months, had diarrheas in the past two weeks, had cough in the past two weeks, and had fever in the past two weeks,

2.7 Operational definitions and variable measurements

Minimum recommended micronutrient intake status: It's the MN intake status among children aged 6–23 months, which was determined by respondents' reports and assessment of intake status. So, there were six options: food rich in VA or iron in the last 24 hours, MNP or iron supplement consumed within the previous seven days, VAS or deworming within the previous six months(19-21, 41). Accordingly, if the respondent reported that the child had eaten' at least one of the minimum recommended MNs, its considered as "Yes"; if the children received none of the minimum recommended MNs, it was considered as "No"(42).

Vitamin A-rich foods: were assessed based on the consumption of seven food groups within the past 24 hours. These groups included: 1) Eggs, 2) Meat (beef, lamb, chicken), 3) Pumpkin, carrots, and squash, 4) Dark green leafy vegetables, 5) Mangoes, papayas, and other Vitamin A-rich fruits, 6) Liver, heart, and other organs, and 7) Fish or shellfish. If the respondent indicated that the child had consumed at least one item from these groups, it was recorded as a 'yes' for Vitamin A-rich food; otherwise, it was recorded as a 'no' (19-21, 41).

Iron-rich foods: were evaluated based on the consumption of four specific food groups within the last 24 hours. These groups included: 1) Eggs, 2) Meat (beef, pork, lamb, and chicken), 3) Liver, heart, and other organs, and 4) Fish or shellfish. If the respondent indicated that the child had consumed at

least one item from these groups, it was recorded as a 'yes' for iron-rich food; otherwise, it was recorded as a 'no' (19-21, 41).

Multiple micronutrient powder: The assessment of multiple micronutrient powders was conducted by asking respondents if their child had received any micronutrient powders in the past seven days (19-21, 41).

Iron supplementation: was evaluated by asking respondents if their child had received iron supplements, such as iron pills, sprinkles with iron, or iron syrup, within the past seven days (19-21, 41).

Vitamin A supplementation (VAS) and deworming for children aged 6–23 months were evaluated by checking if they had received these treatments in the past six months. This assessment was done by reviewing the integrated child health card, which includes immunization and growth monitoring records, as well as obtaining verbal confirmation from the mother(19-21, 41).

Media exposure was assessed as "yes" if they have access to all three media (newsletter, radio, and television) at least once a week, otherwise "no" if they did not have any media exposure(42).

2.8 Data collection tools and technique

2.8.1 Data collection tool and procedure

The questionnaire was prepared in the English language and the tools for data collection are developed from review of guidelines and related literatures (19-21, 41, 42).The contents of the questionnaire included the following three sections: The first section assessed socio-demographic and economic characteristics. The second part deals with obstetric characteristics of the mothers, the third section assessed about child characteristics and common childhood illness and fourth part assessed minimum recommended micronutrient intake which holds 11 questions (19-21, 41, 42). The mothers of the selected children were interviewed, and the data were collected using the Kobo Toolbox, an electronic data collection tool.

A pretested structured questionnaire was used through a face-to-face interview to gather data from mothers. A team of four skilled data collectors, supported by one expert supervisor with backgrounds in public health nutrition, conducted and supervised the data collection process.

2.9 Data quality assurance and control

To ensure quality of data, the questionnaire was translated into the local language (Afar af and Amharic language) by nutrition experts. Finally, before data collection, it was retranslated back to

English to verify consistency. Before starting the actual data collection, one day of training was given for the data collectors and supervisors. A pre-test was conducted on 21 (5%) of the total sample size at Gala.ilu Kebele, which was not selected for study by data collectors, and since internal consistency was adequate, no amendments were made. The reliability of the questionnaires was checked through STATA by reliability index measurement for minimum recommended micronutrient status questions, which showed adequate internal consistency (Cronbach's alpha) of **0.9014.**

The principal investigator and supervisors conducted day-to-day on-site supervision during the whole period of data collection. At the end of each day, the questionnaires were reviewed and checked for completeness and accuracy by the supervisors and investigators. Then corrective modification was made by the principal investigators. Data were checked for completeness, accuracy, clarity, and consistency before being entered into the software. Proper coding and categorization of data was maintained for the quality of the data to be analyzed.

2.10 Data processing, analysis and presentation

The collected data was exported to STATA version 17 for further statistical analysis. Data exploration, editing, and cleaning were undertaken before analysis. Descriptive statistics such as median and interquatile range values for continuous data; percentage, frequency, and tables for categorical data were used. A binary logistic regression analysis was conducted to see factors associated with the minimum recommended micronutrient status and select associated variables with a 95% confidence interval and a P value less than 0.05 for multivariable logistic regression to control the effect of confounders. Then, multivariable analysis was performed to determine the independent predictors of the dependent variable.

Regarding multi-collinearity among independent variables, STATA itself was controlled and checked via variance inflation factor (VIF) and tolerance. Variable VIF greater than 10 and tolerance less than 0.1 were removed in STATA. Accordingly, no multi-collinearity was observed. Final model fitness was checked by the Hosmer and Lemeshow tests, and model adequacy was declared when p-value > 0.05. Accordingly, the final model of Hosmer and Lemeshow showed 0.08. The significance was checked and declared using a p-value less than 0.05 and a 95% confidence interval in the final model. Strength of association was interpreted by using an adjusted odds ratio with a 95% confidence interval.

2.11 Dissemination of result

The findings of this study will be submitted and presented to Samara University, College of Medicine and Health Science, Department of Public Health, as partial fulfillment of a master's degree in nutrition. The final results of this paper will also be submitted to the Aysaita district health office and to all stakeholders, including the Regional Health Bureau, the Zonal Health Department, health institutions, and other non-governmental organizations (NGOs). The result will also be published in reputable journals.

3. Results

3.1 Sociodemographic and socioeconomic characteristics

In this study, all 675 mothers/caregivers with children aged 6-23 months participated, giving a response rate of 100%. The mean age of the mothers was 33.18 (SD ± 4.81). Of these, 351 (52%) were found in the age group of 35–40 years. The majority of the participating children were female, 391 (57.93%). Almost all of the respondents, 675 (100%), were followers of the Muslim religion, and 635 (94.07%) of the study participants belong to the Afar ethnicity. In this study, 614 (90.96%) of the mothers were married and 513 (76%) were housewives.

Remove

Of the mothers, 580 (85.93%) were unable to read and write, followed by read and write education level, which represented 83 (12.3%). Most of the study participants, 491 (72.74%) have four and above family size three children, and about 591 (87.04%) had no monthly income in Ethiopian Birr. The greater majority, 632 (93.63%), of the households had no media exposure (Table 1).

clarify

percent of three children

Table 1: Sociodemographic and economic characteristics of children age 6-23 months and mother/caretakers in the pastoralsit community, Afar Region, Ethiopia, 2024.

Variables	Category	Frequency	Percentage (%)
**Age of mother/caregiver**	18-24	41	6.07
	25-34	283	41.93
	35-40	351	52.00
**Sex of the child**	Male	284	42.07
	Female	391	57.93
**Residence**	Urban	46	6.81
	Rural	629	94.19
**Religion of mother**	Muslim	675	100
**Ethnicity**	Afar	635	94.07
	Amhara	40	5.93

you have taken biological mothers

why you didn't proportionate sampling as urban and rural before data collection?

**Marital status**	Married	614	90.96
	Separated	43	6.37
	Widowed	18	2.67
**Occupation**	Gov't employed	34	5.04
	Housewife	513	76.00
	Merchant	13	1.93
	Pastoralist	115	17.04
**Occupation of the husband/partners**	Daily worker	18	2.67
	Merchant	21	3.11
	Pastoralist	524	77.63
	Farmer	90	13.33
	Gov't employed	22	3.26
**Education status**	Illiterate	580	85.93
	Literate	83	12.30
	Primary education	5	0.74
	Secondary education	7	1.04
**Educational status of the husband/ partners**	Illiterate	528	78.69
	Literate	113	16.84
	Primary education	5	0.75
	Secondary education	17	2.53
	Higher education	8	1.19
**Family size**	≤ 4	184	27.26
	>4	491	72.74
**Monthly income**	≤1000	591	87.56
	1000-5000 1001-4999	67	9.93
	≥5000	17	2.52
**Media exposure**	Yes	43	6.37
	No	632	93.63
**Bank account**	Yes	330	48.89
	No	345	51.11

what types of media? for how long if there is data?

3.2. Obstetric characteristics of the mothers/care givers

The mean age of the mother's first pregnancy was 15.34 (SD ± 2.92). Of these, the majority of them, 580 (85.93%) belonged to the 12–18 age range (<=18 years). In terms of their desire for more children, 625

(92.59%) mothers desire to have more children, while 41 (6.07%) desire no more children over time. Three hundred and eighty-two (56.59%) of the mothers had delivered at home, while 293 (43.41%) of the mothers had delivered in the health institution. About 628 (93.04%) mothers had delivered spontaneous vaginal, while 47 (6.96%) had delivered by cesarean section. Out of the participating specify the PNC mothers, 385 (57.12%) of women had PNC checkups, and 447 (66.22%) stated that they were not pregnant or unsure about their pregnancy status (Table-2).

Table 2**:** Obstetric characteristics of the mothers/care givers of children aged 6–23 months in the pastoralist, Afar Region, Ethiopia, 2024.

Variables	Category	Frequency	Percentage (%)
**Age at 1st pregnancy**	12-18 Better to say <=18	580	85.93
	≥ 19	95	14.07
**Antenatal care follow-up**	Yes	446	66.07
	No	219	32.44
	I don't know	10	1.48 what does it mean?
**Desire for more children**	Undecided	9	1.33
	Wants	625	92.59
	Wants no more	41	6.07
**Place of delivery**	Health facility	293	43.41
	Home	382	56.59
**Mode of delivery**	Cesarean section	47	8.56
	Spontaneous vaginal delivery	628	93.04
**Post natal check up**	Yes	385	57.12
	No	289	42.88
**Current pregnancy status**	Non pregnant or unsure	447	66.22
	Pregnant	228	33.78

3.3 Child characteristics and common childhood illness

Participated children had a median age of 8 months with an interquartile range (IQR = 7-12 months). Among the included children, about one hundred twenty-nine (19.11%) had exclusive breast-feeding status. Out of the participating children, 366 (52.32%) had diarrhea oprationalize in the past two weeks, and 322 (47.70%) of them had cough in the past two weeks. Regarding fever, remove 257 (38.07%) of children had fever in the past two weeks (Table-3).

Table 3: Child characteristics and common childhood illness of children aged 6–23 months at at Aysaita district, Afar Region, Ethiopia, 2024.

Variables	Category	Frequency	Percentage (%)
Current age of the child (months)	6-11 months	469	69.48
	12-23 months	206	30.52
Exclusive breastfeeding status	Yes	129	19.11
	No	546	80.89
Had diarrhoea in the past two weeks	Yes	366	54.22
	No	309	45.78
Had cough in the past two weeks	Yes	322	47.70
	No	353	52.30
Had fever in the past two weeks	Yes	257	38.07
	No	418	61.93

3.4 Micronutrient intake status of the childrens aged 6-23 months

According to this study, 236 (34.96%) children between the ages of 6 and 23 months had received at least one minimum recommended micronutrient intake status (34.96%; 95% CI: 31.45, 38.64%) (Fig. 3). Overall, 65.04% (95% CI: 61.35, 68.54) of children between 6 and 23 months old hadn't received any MNs from the recommended sources. Thirteen point four eight (95% CI: 11.10-16.27) of the children received iron supplements, 12.59% (95% CI: 10.29-15.32) of the children received multiple MNP within the last seven days, and 22.52% (95% CI: 19.51-25.83) of the children received VAS within the previous six months. About 24.30% (95% CI: 21.20–27.68) of the children received deworming medication within the last six months. Merely 27.41% (95% CI: 24.16–30.90) of the children had meals rich in VA, and 21.33% (95% CI: 18.40–24.59) had food rich in iron in the previous 24 hours (Table 4).

Table 4: Minimum recommended micronutrient intake status among children aged 6–23 months in the pastoralist community, Afar Region, Ethiopia, 2024.

No	Food groups and supplementations	Received status		
		Response	Frequency	Percent (%)
	**1. Vitamin A-rich foods within 24 hours**			
**1.1**	Did your child eat those vitamin-A-reached and deep yellow-colored fruits like ripe mango, papaya, (within 24 hours) or pure juice made from these fruits?	Yes	119	17.63
		No	556	82.37
**1.2**	Did your child eat eggs yesterday (within 24 hours) at home or outside?	Yes	117	17.33
		No	558	82.67
**1.3**		Yes	100	14.81

	Did your child eat meat (beef, lamb, chicken, etc.) yesterday (within 24 hours) at home or outside?	No	575	85.19
**1.4**	Did your child eat those vegetables, pumpkin, carrots, and squash yesterday (within 24 hours) at home or outside?	Yes	116	17.19
		No	559	82.81
**1.5**	Did your child eat those other dark green leafy vegetables yesterday (within 24 hours) at home or outside?	Yes	102	15.11
		No	573	84.89
**1.6**	Did your child eat meat from the liver, heart, and other organs yesterday (within 24 hours) at home or outside?	Yes	102	15.11
		No	573	84.89
**1.7**	Did your child eat meat from fish or shellfish yesterday (within 24 hours) at home or outside?	Yes	79	11.70
		No	596	88.30
	Total Vitamin A-rich foods within 24 hours	***Yes***	***185***	***27.41***
		No	***490***	***72.59***
	**2. Iron-rich foods**			
**2.1**	Did your child eat eggs yesterday (within 24 hours) at home or outside?	Yes	117	17.33
		No	558	82.67
**2.2**	Did your child eat meat (beef, lamb, chicken, etc.) yesterday (within 24 hours) at home or outside?	Yes	100	14.81
		No	575	85.19
**2.3**	Did your child eat meat from the liver, heart, and other organs yesterday (within 24 hours) at home or outside?	Yes	102	15.11
		No	573	84.89
**2.4**	Did your child eat meat from fish or shellfish yesterday (within 24 hours) at home or outside?	Yes	79	11.70
		No	596	88.30
	Total Iron-rich foods consumptions	***Yes***	***144***	***21.33***
		No	***531***	***78.67***
**3**	Did your child receive ***multiple micronutrient powders*** in the past seven days?	Yes	85	12.59
		No	590	87.41
**4**	Did your child receive ***iron supplements*** within seven days (iron pills, sprinkles with iron, or iron syrup in the previous seven days)?	Yes	91	13.48
		No	584	86.52
**5**	Did your child receive ***Vitamin A supplementation*** within six months (by reviewing the integrated child health card, immunization card, or growth monitoring history) otherwise from the mother's verbal response?	Yes	79	22.52
		No	523	77.48
**6**	Did your child receive ***deworming medication*** in the six months (by reviewing the integrated child health card, immunization card, or growth monitoring history) otherwise from the mother's verbal response?	Yes	164	24.30
		No	511	75.70
	Overall minimum recommended micronutrient intake status	No	439	65.04
		Yes	236	34.96
		give some brief explanation		

3.4 Factors associated with minimum recommended micronutrient intake status

Based on bivariate logistic regression, being a male child, media exposure, having a postnatal checkup, having exclusive breast feeding, spontaneous mode of delivery, and income category 500-5000 were significantly associated with the minimum recommended micronutrient intake status, and all were transferred to multivariable logistic regression analysis to control the effect of confounders. However, in the multivariable logistic regression analysis, being a male child, media exposure, having exclusive breast feeding, spontaneous vaginal delivery, and income category 500-5000 were identified as predictors of minimum recommended micronutrient intake status ($p < 0.05$) (Table 5).

Accordingly, findings from multivariable logistic regression showed that the odds of receiving minimum recommended micronutrient intake were 2.42 times higher for children whose mothers gave birth by spontaneous vaginal than for children whose mothers gave birth by cesarian section (AOR: 2.42, 95% CI: 1.12, 5.25). The odds of receiving minimum recommended micronutrient intake among children whose mothers had been exposed to media were 2.09 more likely as compared to those not exposed to media (AOR = 2.09; 95% CI: 1.16, 3.75). Being a male child was 1.98 times [AOR = 1.98; 95% CI: 1.41, 2.78] more likely to receive the minimum recommended micronutrient intake as compared to those of females. Again, the odds of receiving minimum recommended micronutrient intake were 4.53 times higher for those children who had exclusive breast feeding than those who had no exclusive breast feeding (AOR: 4.53, 95% CI: 2.51, 8.19). Furthermore, households that had a monthly income category of 1000-5000 were 1.99 more likely to receive the minimum recommended micronutrient intake than those who had no monthly income category (AOR: 1.99, 95% CI: 1.17, 3.39).

Table 5: Bivariable and multivariable analysis of factors associated with minimum recommended micronutrient intake status among children aged 6–23 months living in the pastoralsit comminty, Afar Region, Ethiopia, 2022

Variables	Category	Yes	No	COR (95%CI)	AOR (95%CI)	P-value for AOR
**Sex of the child**	Male	124(43.66)	160(56.34)	1.93(1.40, 2.66) *	***1.98(1.41, 2.78)***	***0.000***
	Female	112(28.64)	279(71.36)	1	1	
**Media exposure**	Yes	35(58.33)	25(41.67)	2.91(1.69, 4.99) *	***2.09(1.16, 3.75)***	***0.014***
	No	199(32.46)	414(67.54)	1	1	
**Post natal check up**	Yes	157(40.78)	228(59.22)	1.83(1.31, 2.54) *	1.17(0.81, 1.70)	0.38
	No	79(27.34)	210(72.66)	1	1	

**Exclusive breast feeding**	Yes	220(40.29)	326(59.71)	4.76(2.74, 8.26) *	***4.53(2.51, 8.19)***	***0.00****
	No	16(12.40)	113(87.60)	1	1	
**Mode of delivery**	Spontaneous Vaginal	226 (35.99)	402(64.01)	2.08(1.01, 4.26) *	***2.42(1.12, 5.25)***	***0.024****
	Cesarian section	10(21.28)	37(78.72)	1	1	
**Income category**	≤1000	198(33.50)	393(66.50)	1	1	
	1000-5000	38(45.24)	46(54.76)	1.63(1.03, 2.60) *	***1.99(1.17, 3.39)***	***0.01****

4. Discussion

The Ethiopian national guidelines for the prevention and control of micronutrient deficiency state that micronutrients must be given to all children(43). Our study, however, showed that only 34.96% (95% CI: 31.45, 38.64%) of 6 to 23-month-old'children had received any of the minimum recommended MN sources in the study area. Based on the findings, the minimum recommended micronutrient intake status in the study area is far from meeting National Nutrition Program (NNP II) 2016–2020 targets, indicating that a significant percentage of its 6- to 23-month-old children do not consume the recommended amount of micronutrients as per national guidelines.

This finding is comparatively higher than studies conducted in Ethiopia, 27.6% (44). However, the finding is comparatively lower than to previous studies conducted in Ethiopia 62.7% (42), sub-Saharan Africa 73.99% (40), Rwanda (68%), Burundi (78%) (45), and India (58.1%) (46). The possible discrepancy for this prevalence may be justified by the fact that variation in dietary intake across regions due to cultural, availability, and accessibility could be a probable explanation. Additionally, each geographic region has a different staple food due to differences in literacy, food insecurity, traditions, and beliefs.

After adjusting the odd ratio in the final multivariable logistic regression, being a male child, media exposure, having exclusive breast feeding, spontaneous vaginal delivery, and income category 1000-5000 were significantly associated factors with the minimum recommended micronutrient intake status among children aged 6–23 months.

Children whose mothers were exposed to media were more likely to consume minimum recommended

not necessery, also for others too .

micronutrients compared to those whose mothers were not. This finding aligns with studies conducted in Ethiopia (32, 47, 48), Nigeria (49), Indonesia (50), and Nepal (51). The influence of national radio and television promotions on child nutrition-related advertisements likely contributed to this outcome (47).

this is conclusion

This suggests that creating media campaigns highlighting the importance of micronutrient intake for children and promoting the recommended amounts can significantly improve children's micronutrient consumption. Therefore, the government should consider expanding current programs that use mass media to promote child nutrition practices, thereby enhancing mothers' awareness of the importance of micronutrients. this is recommendation

This study found that children whose mothers gave delivery by spontaneous vaginal delivery had higher odds of minimum recommended micronutrient intake compared to those whose mothers gave delivery by cesarian section. One possible explanation is that mothers who gave birth by cesarean section initiate late breast feeding, and infants born via cesarean section tend to experience more distress and require specialized feeding practices, which can impact the length of breastfeeding and child gastrointestinal mucosa, which impairs gastrointestinal absorption. Additionally, cesarean deliveries are linked to the use of formula supplementation, disrupting exclusive breastfeeding and raising the likelihood of early weaning, developmental delay, and malnutrition (52, 53).

This study also found that children who had exclusive breast feeding had higher odds of minimum recommended micronutrient intake compared to those whose mothers did not. This might be due to the fact that exclusive breastfeeding has been shown to effectively support the optimal growth of young children (54). Moreover, this study found that being a male child had higher odds of minimum recommended micronutrient intake as compared to those of females. This finding is similar to those of a study conducted in Ethiopia in Diredawa city (55) and Haramaya town (56). This might be due to the traditional influence on male sex preference in Ethiopian populations, and they gave attention to the feeding of a variety of food items to their male child.

This study also revealed that children whose households had higher monthly income were more likely to intake the minimum recommended micronutrient intake than households that had a monthly income less than 1000 ETB. This finding is consistent with a study conducted in Ethiopia and a secondary data analysis of the Nepalese Demographic and Health Survey(51). Similarly, the 2011 Ethiopian DHS reported that children from the wealthiest families were more likely to consume four or more food groups (57). This could be because families with higher monthly incomes can afford a more diverse diet for their children compared to those with lower incomes.

As a limitation, this study may not demonstrate a causal relationship between predictors and minimum recommended micronutrient intake status because of its design. And also, social desirability bias might occur.

5. Conclusions and recommendations

remove not necessery

In conclusion, the study reveals that despite national guidelines advocating for micronutrient supplementation for all children, only 34.96% of children aged 6 to 23 months in the study area received the minimum recommended micronutrients. This falls short of the National Nutrition Program (NNP II) targets, indicating a significant gap in micronutrient intake among young children. Being a male child, media exposure, exclusive breastfeeding, spontaneous vaginal delivery, and household income were significantly associated with the minimum recommended micronutrient intake for children aged 6-23 months. the conclusion need revision

Recommendations for the health bureau: write in one paragraph (make it short)

- Expand and intensify media campaigns focused on child nutrition to raise awareness and educate parents about the importance of micronutrient intake. Because mothers exposed to media are more likely to ensure their children to meet the minimum recommended micronutrient intake.
- In addition, utilizing national radio and television to broadcast child nutrition-related advertisements can significantly improve awareness and practices. your specific media was not mentioned
- Furthermore, implement educational programs to address cultural biases and ensure equal feeding practices for both male and female children. Male children are more likely to meet micronutrient intake recommendations, possibly due to cultural preferences. Therefore, ensuring equal attention to the nutrition of female children is crucial. how do you know there were not equal feeding were not practiced?

Recommendations for all health professionals:

- Advocate mothers' educations regarding exclusive breastfeeding for the first six months of life, as it supports optimal growth and micronutrient intake. This should include community-based programs and support groups to encourage and assist mothers in exclusive breastfeeding practices.
- Additionally, encourage and support spontaneous vaginal deliveries where medically feasible as it facilitates earlier initiation of breastfeeding and fewer complications compared to cesarean sections.

Recommendations for further researchers: Furthermore, to establish a causal-effect relationship between minimum recommended micronutrient intake status and its contributing factors, additional longitudinal studies should be carried out.

Non-governmental organization that can provide financial and nutritional support: Provide financial support and income generation resources to low-income families to improve their ability to afford diverse and nutritious diets. Therefore, economic support can help families provide a more varied diet. your recommendation should be based on your findings. remove all irrelevant recommendations

List of Abbreviations and Acronyms

ANC: Antenatal Care

AOR: Adjusted Odds Ratio

CI: Confidence Interval

COR: Crude Odds Ratio

CSA: Central Statistical Agency

EDHS: Ethiopian Demographic and Health Survey

FAO: Food and Agriculture Organizations

FMoH: Federal Ministry of Health

MDD: Minimum Dietary Diversity

MN: Micronutrients

MNP: Multiple Micronutrient Powder

NGO: Non-Governmental Organization

PNC: Postnatal Care

SDG: Sustainable Development Goals

VA: Vitamin A

VAS: Vitamin A supplements

WHO: World Health Organization

Funding statements

The author(s) received no financial support for the research, authorship, and/or publication process of this article.

Acknowledgments

The authors would like to thank Samara University, College of Medicine and Health Science, regional health office, zone health office, woreda health bureau and administrative office, Woreda health extension worker, data collectors, supervisors, and study participants for their cooperation in the study.

Oral informed consents/patient consents

Each study participant was adequately informed by their data collectors about the purpose, contents of the study, method, anticipated benefit and risk of the study, and the right to withdraw from the study at any time. An ***oral informed consent*** was obtained from the study participants before data collection during study time. In addition, participants were assured about the aim, possible risk, if any, and confidentiality of the study using their own local language.

Trial registration number/date: Not applicable

Grant number: Not applicable

Ethical Statements

Ethical approval was obtained from the ***Institutional Ethical Review Committee (IERC) of Samara University, College of Medicine and Health Science, with reference number (Ref. No.: SU/CMHS/IERC/814/2016)***. Following the approval by IERC, official letters of cooperation were written to the Aysaita district health administration office, and in turn, the woreda health administration office wrote letters to each selected kebeles and village's administration office in order to get permission and cooperation. ***Oral informed consent*** was obtained from the parents or guardians of participants after explaining the purpose and objective of the study. To ensure confidentiality, the names of respondents were replaced by code numbers. Participants were given the chance to ask any doubt about the study and made free to refuse or stop the interview at any moment they wanted.

Conflict of interest statement: The authors declared that they have no conflicts of interest.

Data availability statement: Data availability for this article is available up on reasonable request from the principal investigators.

Authors' Contributions

Abdulkerim Hassen Moloro: Designed the study, wrote the introduction and methods, performed the analysis and interpretation of data, drafted the manuscript, and approved it.

Ali Mohammed Ibrahim, Dereje Desalegn Boshe and Yeshimebet Ali Dawed*:* assisted in designing the study and methods, came up with the first draft, interpretation, prepared figures, and critically revised the manuscript. All authors read and approved the final manuscript.

References

1. Martorell R. Improved nutrition in the first 1000 days and adult human capital and health. American Journal of Human Biology. 2017;29(2):e22952.

2. Girma W, Genebo T. Determinants of nutritional status of women and children in Ethiopia. 2002.

3. Black RE, Victora CG, Walker SP, Bhutta ZA, Christian P, De Onis M, et al. Maternal and child undernutrition and overweight in low-income and middle-income countries. The lancet. 2013;382(9890):427-51.

4. FMo H. National nutrition strategy 2008.

5. Ahmed T, Hossain M, Sanin KI. Global burden of maternal and child undernutrition and micronutrient deficiencies. Annals of Nutrition and Metabolism. 2013;61(Suppl. 1):8-17.

6. Kiani AK, Dhuli K, Donato K, Aquilanti B, Velluti V, Matera G, et al. Main nutritional deficiencies. Journal of preventive medicine and hygiene. 2022;63(2 Suppl 3):E93.

7. Black MM. Micronutrient deficiencies and cognitive functioning. The Journal of nutrition. 2003;133(11):3927S-31S.

8. Tulchinsky TH. Micronutrient deficiency conditions: global health issues. Public health reviews. 2010;32:243-55.

9. Hassen HY, Ali JH, Gebreyesus SH, Endris BS, Temesgen AM. National incidence, prevalence and disability-adjusted life years (DALYs) of common micronutrient deficiencies in Ethiopia from 1990 to 2017: estimates from the global burden of diseases study. Global health action. 2020;13(1):1776507.

10. FMo. H. Guidelines for the prevention and control of Micronutrient deficiencies in Ethiopia Federal Ministry of Health. . 2016.

11. D. M. Ethiopia Demographic and Health Survey 2011, Final Report. Calverton, MD. 2011.

12. ICF. CSACEa. Ethiopia Demographic and Health Survey 2016. Addis Ababa, Ethiopia, and Rockville, Maryland, USA: CSA and ICF. . 2016.

13. Institute. TEPH. Ethiopian National Micronutrent Survey Report 2016.

14. Demissie T, Ali A, Mekonen Y, Haider J, Umeta M. Magnitude and distribution of vitamin A deficiency in Ethiopia. Food and nutrition bulletin. 2010;31(2):234-41.

15. Organization WH. Global prevalence of vitamin A deficiency in populations at risk 1995-2005: WHO global database on vitamin A deficiency. 2009.

16. Lander RL, Enkhjargal T, Batjargal J, Bailey KB, Diouf S, Green TJ, et al. Multiple micronutrient deficiencies persist during early childhood in Mongolia. Asia Pac J Clin Nutr. 2008;17(3):429-40.

17. Ahmed F, Prendiville N, Narayan A. Micronutrient deficiencies among children and women in Bangladesh: progress and challenges. Journal of nutritional science. 2016;5:e46.

18. Sheehy T, Carey E, Sharma S, Biadgilign S. Trends in energy and nutrient supply in Ethiopia: a perspective from FAO food balance sheets. Nutrition journal. 2019;18:1-12.

19. Guideline W. use of multiple micronutrient powders for point-of-use fortification of foods consumed by infants and young children aged 6–23 months and children aged 2–12 years. World Health Organization: Geneva, Switzerland. 2016.

20. Dary O, Hurrell R. Guidelines on food fortification with micronutrients. World Health Organization, Food and Agricultural Organization of the United Nations: Geneva, Switzerland. 2006;2006:1-376.

21. Joint F. Human vitamin and mineral requirements. 2002.

22. Saldanha LS, Buback L, White JM, Mulugeta A, Mariam SG, Roba AC, et al. Policies and program implementation experience to improve maternal nutrition in Ethiopia. Food and nutrition bulletin. 2012;33(2_suppl1):S27-S50.

23. Organization. WH. Global nutrition targets 2025: anaemia policy brief: World Health Organization; . 2014.

24. Canton H. Food and agriculture organization of the United Nations—FAO. The Europa directory of international organizations 2021: Routledge; 2021. p. 297-305.

25. Organization WH. Levels and trends in child malnutrition child malnutrition: UNICEF/WHO/World Bank Group Joint Child Malnutrition Estimates: Key findings of the 2023 edition: World Health Organization; 2023.

26. Ethiopia. FDRo. Food and Nutrition Policy 2018. 2018.

27. Ethiopia. FDRo. National nutrition program 2016–2020 2016. 2016–2020 2016.

28. Ethiopia. FDRo. Seqota Declaration 2015. 2015.

29. Ephi I. Ethiopian public health Institute (EPHI)[Ethiopia] and ICF. Ethiopia mini demographic and health survey. 2019.

30. Kassie GA, Gebrekidan AY, Enaro EY, Asgedom YS. Minimum acceptable dietary intake among children aged 6–23 months in Ethiopia: A systematic review and meta-analysis. PloS one. 2023;18(6):e0287247.

31. Mohammed SH, Habtewold TD, Arero AG, Esmaillzadeh A. The state of child nutrition in Ethiopia: an umbrella review of systematic review and meta-analysis reports. BMC pediatrics. 2020;20:1-10.

32. Dangura D, Gebremedhin S. Dietary diversity and associated factors among children 6-23 months of age in Gorche district, Southern Ethiopia: Cross-sectional study. BMC pediatrics. 2017;17:1-7.

33. Mulat E, Alem G, Woyraw W, Temesgen H. Uptake of minimum acceptable diet among children aged 6–23 months in orthodox religion followers during fasting season in rural area, DEMBECHA, north West Ethiopia. BMC nutrition. 2019;5:1-10.

34. Iannotti L, Lesorogol C. Dietary intakes and micronutrient adequacy related to the changing livelihoods of two pastoralist communities in Samburu, Kenya. Current Anthropology. 2014;55(4):475-82.

35. Bach A, Gregor E, Sridhar S, Fekadu H, Fawzi W. Multisectoral integration of nutrition, health, and agriculture: implementation lessons from Ethiopia. Food and nutrition bulletin. 2020;41(2):275-92.

36. Haile D, Biadgilign S, Azage M. Differentials in vitamin A supplementation among preschool-aged children in Ethiopia: evidence from the 2011 Ethiopian Demographic and Health Survey. Public health. 2015;129(6):748-54.

37. Gebre-Egziabhere T. Emerging Regions in Ethiopia: Are they catching up with the rest of Ethiopia? Eastern Africa Social Science Research Review. 2018;34(1):1-36.

38. Stark J, Terasawa K, Ejigu M. Climate change and conflict in pastoralist regions of Ethiopia: mounting challenges, emerging responses. Conflict Management and Mitigation (CMM) Discussion Paper. 2011;4.

39. Agency. FDRoECS. Population Projection of Ethiopia for All Regions At Wereda Level from 2014 – 2017. Archived from the original on 6 June 2018. Retrieved 4 June 2018

2014 – 2017,.

40. Engidaw MT, Gebremariam AD, Tiruneh SA, Tesfa D, Fentaw Y, Kefale B, et al. Micronutrient intake status and associated factors in children aged 6–23 months in sub-Saharan Africa. Scientific reports. 2023;13(1):10179.

41. Tabacchi G, Wijnhoven TM, Branca F, Román-Vinas B, Ribas-Barba L, Ngo J, et al. How is the adequacy of micronutrient intake assessed across Europe? A systematic literature review. British journal of nutrition. 2009;101(S2):S29-S36.

42. Gebremedhin T, Aschalew AY, Tsehay CT, Dellie E, Atnafu A. Micronutrient intake status and associated factors among children aged 6–23 months in the emerging regions of Ethiopia: A multilevel analysis of the 2016 Ethiopia demographic and health survey. PloS one. 2021;16(10):e0258954.

43. FMO. H. Guidelines for the prevention and control of Micronutrient deficiencies in Ethiopia Federal Ministry of Health. 2016.

44. Fetene SM, Negash WD, Amare T, Belachew TB, Addis B, Asmamaw DB, et al. Suboptimal micronutrient intake among children aged 6 to 23 months in Ethiopia: Further analysis of the demographic and health survey. PloS one. 2024;19(7):e0305232.

45. Custodio E, Herrador Z, Nkunzimana T, Węziak-Białowolska D, Perez-Hoyos A, Kayitakire F. Children's dietary diversity and related factors in Rwanda and Burundi: a multilevel analysis using 2010 Demographic and Health Surveys. PloS one. 2019;14(10):e0223237.

46. Semba RD, de Pee S, Sun K, Campbell AA, Bloem MW, Raju V. Low intake of vitamin A–rich foods among children, aged 12–35 months, in India: association with malnutrition, anemia, and missed child survival interventions. Nutrition. 2010;26(10):958-62.

47. Beyene M, Worku AG, Wassie MM. Dietary diversity, meal frequency and associated factors among infant and young children in Northwest Ethiopia: a cross-sectional study. BMC public health. 2015;15:1-9.

48. Aemro M, Mesele M, Birhanu Z, Atenafu A. Dietary diversity and meal frequency practices among infant and young children aged 6–23 months in Ethiopia: a secondary analysis of Ethiopian demographic and health survey 2011. Journal of nutrition and metabolism. 2013;2013(1):782931.

49. Aremu O, Lawoko S, Dalal K. Childhood vitamin A capsule supplementation coverage in Nigeria: a multilevel analysis of geographic and socioeconomic inequities. The Scientific World Journal. 2010;10(1):1901-14.

50. Sekartaji R, Suza DE, Fauziningtyas R, Almutairi WM, Susanti IA, Astutik E, et al. Dietary diversity and associated factors among children aged 6–23 months in Indonesia. Journal of Pediatric Nursing. 2021;56:30-4.

51. Joshi N, Agho KE, Dibley MJ, Senarath U, Tiwari K. Determinants of inappropriate complementary feeding practices in young children in Nepal: secondary data analysis of Demographic and Health Survey 2006. Maternal & child nutrition. 2012;8:45-59.

52. Cato K, Sylven SM, Lindbäck J, Skalkidou A, Rubertsson C. Risk factors for exclusive breastfeeding lasting less than two months—identifying women in need of targeted breastfeeding support. PloS one. 2017;12(6):e0179402.

53. Chen C, Yan Y, Gao X, Xiang S, He Q, Zeng G, et al. Influences of cesarean delivery on breastfeeding practices and duration: a prospective cohort study. Journal of Human Lactation. 2018;34(3):526-34.

54. WHO. Exclusive Breastfeeding for Optimal Growth, Development and Health of Infants. e-Library of Evidence for Nutrition Actions (eLENA) 2019. 2019.

55. Sema A, Belay Y, Solomon Y, Desalew A, Misganaw A, Menberu T, et al. Minimum dietary diversity practice and associated factors among children aged 6 to 23 months in Dire Dawa City, Eastern Ethiopia: a community-based cross-sectional study. Global pediatric health. 2021;8:2333794X21996630.

56. Berhe Gebremichael GE, Assefa N. Dietary diversity practice and associated factors among infants and young children in Haramaya town, Ethiopia. Int J Public Health Sci. 2017;6(3):243-50.

57. Demographic N. Health survey 2011. Central Statistical Agency Addis Ababa Ethiopia ICF International Calverton, Maryland, USA. 2012;2016(1).

Copy of the ethical review committee

Click here to access/download

Supporting Information

Copy of Ethical review committee letter.pdf
