# Supplementary material for: Minimum recommended micronutrient intake status and associated factors among pastoralist children aged 6–23 months in Aysaita district, Afar Region, Ethiopia, 2024: A community based cross-sectional study
Source: PLoS One. 2025 Oct 28;20(10):e0334736. doi: 10.1371/journal.pone.0334736 (PMC12561942; doi:10.1371/journal.pone.0334736)
Supplement: Attachment — Submitted filename: Review comments_Minimum Recommended Micronutrient Intake.pdf [file pone.0334736.s004.pdf]

Manuscript: PONE-D-24-55242

Title: Minimum Recommended Micronutrient Intake Status and Associated Factors among Children Aged 6-23 Months in Pastoralist Community of Afar Region, Ethiopia, 2024 : Community Based Cross-sectional Study

Review comments

Firstly, thank you very much for providing the opportunity to review this manuscript. Second, I will list out all the important comments to be incorporated within the manuscript for a better research output. However, the article has no line number for providing comments easily for authors. Therefore, the authors need to use standard page numbering of articles and line number while resubmitting the article which would be important for the second round of comments.

Authors order

1. Order the Authors in terms of Authors contribution

ABSTRACT

2. Page 11: line 19. Write the currency of the income category 500-5000 whether it was USD or ETB?
3. Page 11: line 22-23. The Authors need to rewrite all the conclusion and recommendations.
   3.1 Conclude and recommend based on your research findings and your study groups (6-23 months).
   3.2 The study revealed over one-third of the children received the minimum recommended micronutrient intake. Make it measurable, clear and easily understandable for readers.
   3.3 Use the updated national nutrition program policies and strategies of the country rather than using the outdated National Nutrition Program (NNP II) 2016-2020.

Introduction

4. Page 12: line 5-6: Use the sub title either of the word introduction or background. It is preferred to use the word introduction rather than the word background. It is more formal.
5. Page 12: line 8-10: However, during this time, children are highly susceptible to undernutrition due to factors like inadequate dietary intake, limited access to food, nutritional taboos, and infectious diseases (2). The Authors list out the factors contributed for undernutrition with identifying the immediate cause of malnutrition. What about the underlining and basic causes that most commonly contribute for malnutrition? It would be better if the authors add those points with their citations.

Methods

Study design, setting and periods

6. Page 14: line 6: Afar National Regional State is one of the eleven regional states of Ethiopia. The Authors need to update it.
7. Page 14: line 10-13: The last administrative study was Districts and Kebeles. The Authors need to discuss districts available in each zones in detail.
8. Page 14: line 14-15: …and a total population projection for 2017 was 1,812,002, consisting of 991,000 men and 821,002 women (39). The Authors need to use the most recent population of the region.
9. Page 14: line 14-15: For this study, Assayta district was selected randomly, which was found in Awsi Rasu (Administrative Zone 1) within the Afar Region, Ethiopia. How one district (Assayta district-11 kebele) represents the entire Afar region? How would your study have generalized to the whole 6-23months children living in Afar region? Even how do you think that Assayta district findings will be generalized to Awsi Rasu (Administrative Zone 1)? In research, at least 30% of the target population should be sample to be generalized. The Authors should see this as a serious issue and your research title would be revised.
10. Page 14: line 4-23: The study was conducted in pastoralist communities of Afar Region. However, I can't see any thing explained about the pastoralist communities. Does all the population of the Afar region totally (100%) pastoralist communities? Make it clear for the readers.
11. Page 14: line 20-22: The district is administratively divided into 11 kebeles. According to the Aysaita District Administration, the total human population of the district is 61961. Among the total population of the district, 12392 households have children aged 6–23 months. Cite your reference here.
12. Page 14: line 27-29: Source population**,** All children aged 6-23 months and mothers/caregivers residing in pastoralist communities of the Afar region were source populations. Did the mothers/caregivers were also your study population?
13. Page 15: line 1-2: Your study population was All children 6-23 months old and who are residents of the selected kebeles in the selected district. How those children 6-23 months old systematically selected represent all children 6-23 months old residing in Afar region?

14. Page 15: line 1-2: In your Exclusion criteria, Children living with others than their mothers or caregivers were excluded from the analysis. I think children living with others than their mothers or caregivers were in appropriately excluded from the study. Because, children living with others than their mothers or caregivers would be one factor for Minimum Recommended Micronutrient Intake.
15. Page 15: line 13-14: Why you used the OpenEpi online software version 3.1 instead of using population determination formula? It could be better if you were used the OpenEpi online software for determining the sample size for factors associated with the Minimum Recommended Micronutrient Intake status.
16. Page 15: line 13-19: I didn't see the sample size determination for factors associated with the Minimum Recommended Micronutrient Intake status. How do you think that the dependent variable sample size was representing the factors for your dependent variable?
17. Page 15: line 15: First, Zone One was randomly selected from the six zones in the Afar region. How this one zone will represent the other 5 zones/6 zones? It is a major issue to be solved.
18. Page 17: line 15: MN. Always use the first abbreviations together with full phrase.

Dissemination of result

19. Page 19-20: line 1-5: Remove all dissemination of the result. It is not the part of the article rather than during your research proposal.

Result

20. Page 21: Table 1.: Family size, what is your evidence for the categorizing Family size with ≤ 4 and >4 only?
21. Page 21: Table 1.: Monthly income, does this income per month or annum? what is the currency of income? Which one is correct? USD or ETB? Much better and easily understandable if explained in USD for general readers but not only for Ethiopians.
22. Page 21: line 31-32: It says…Of these, the majority of them, 580 (85.93%) belonged to the 12–18 age range. The age range written here-12-18 is quite different from the age group categorized in table 1, page 21: line 21-23. Please take corrective actions.
23. Page 24: line 15: Relace the word "Again" with in "addition"
24. Page 26: The monthly income category in table 1 and 5 are quite different. Therefore, please update it.

25. Page 26: Table 5, the Asterisk (*) listed out in table 5 is not explained in foot note**.**

Discussion

26. Page 26: line 20-21: The possible reasons should be cited from credible studies. And try to cite research evidence of why these variables are associated, not just supposition. The comment works through out the discussion.
27. Page 26: line 16-21: Provide your reasons individually why your research finding was higher than X studies and why your research finding were lower than X studies based on relevant references/studies/citation. It works for all discussions.
28. Page 27: line 3-5: It that explanation of evidence or your recommendation. If it is your recommendation, please put it in Conclusion and recommendations component of your article.

References

29. Page 31-34: Use the selected/used references in your article other useless references should be removed.

Annex

30. Add the questionaries used for data collection as a supplementary material.

Overall comment

This study is well-conducted, and the researchers are commended for their work thorough the title of Minimum Recommended Micronutrient Intake Status and Associated Factors among Children Aged 6-23 Months in Pastoralist Community of Afar Region, Ethiopia, 2024 : Community Based Cross-sectional Study. The study is effectively structured, with a clear identification of the impact of Minimum Recommended Micronutrient Intake Status with well thought recommendations for public health interventions. However, to enhance its credibility and ensure clear communication of the findings, the researchers should address the minor comments and suggestions provided above. Once these revisions are made, the study will be ready for publication and will provide valuable insights for controlling Micronutrient deficiency in your study area and the country as well.
